# Supplementary material for: What about tocilizumab? A retrospective study from a NYC Hospital during the COVID-19 outbreak
Source: PLoS One. 2021 Apr 8;16(4):e0249349. doi: 10.1371/journal.pone.0249349 (PMC8031323; doi:10.1371/journal.pone.0249349)
Supplement: Attachment — Submitted filename: Response to Reviewers 2-18-21.docx [file pone.0249349.s002.docx]

Monica Mehta, PharmD, MPH

Lawrence Purprua, MD

Thomas McConville, MD

February 17, 2021

Re: Response to Reviewers

Dear Dr. Zivkovic and other editors at PLOS ONE,

Thank you for reading and reviewing our paper! As per your instructions, we have addressed reviewer comments below. Additionally, we have made edits to our paper using track edits. These changes are partly intended to address reviewer comments, but also update the discussion section since several tocilizumab papers have been published in the previous 3-months while our manuscript was being reviewed.

Feel free to reach out with any further edits or questions.

Sincerely,

Monica, Lawrence, and Thomas

NewYork-Presbyterian Hospital

Columbia University Irving Medical Center

New York, New York

--

Reviewer 2 had two questions:

1. How was information regarding receipt of tocilizumab prior to hospitalization obtained? Was it confirmed that patients were taking tocilizumab? Could this be a limitation to the study?

Response: Upon chart review, we did not identify any patients who received tocilizumab prior to admission in our electronic medical record. We also excluded patients with active malignancy; therefore, use of tocilizumab for cytokine release syndrome is highly unlikely. Moreover, that would be given in the inpatient setting. It is remotely possible that one of our study patients received tocilizumab as an outpatient at a different medical system. We can include a sentence in the discussion section making this point. Added, “Though no doses of tocilizumab prior to admission were documented in our electronic health record; there is a remote possibility that previous doses were given outside our hospital system for rheumatoid arthritis.”

2. What medication were given to COVID-19 patients along with tocilizumab? If the authors have collected data concerning each individual inpatient prescription chart beyond the details of their receipt. All participants received what standardized COVID-19 treatment protocol along with tocilizumab in regular medications?

Response: Great question. See here with a table describing concurrent medications for our cohort. Because of uncertainty around implications of these medications, we did not include them in the final paper. At this point, the only medication with a clear mortality benefit is dexamethasone (or other corticosteroid), but patients receiving steroids were excluded from our study in order to understand the benefit of tocilizumab alone. We added a sentence describing remdesivir use between groups in our paper. Added, “Only 6.1% of patients in the tocilizumab group and 5.9% in the control group received remdesivir because it was only available by compassionate use during the study period.”

| Characteristics of Patients Receiving or Not Receiving Tocilizumab | | | |
| --- | --- | --- | --- |
|  |  | Tocilizumab  N = 33 | Control  N = 74 |
| Home medications | ACE Inhibitors | 11 (33.3) | 18 (24.3) |
|  | Corticosteroids | 2 (6.1) | 3 (4.1) |
|  | Warfarin | 4 (12.1) | 6 (8.1) |
|  | Statin | 9 (27.3) | 22 (29.7) |
| COVID-19 medications | Remdesivir | 2 (6.1) | 4 (5.9) |
|  | Hydroxychloroquine | 25 (75.5) | 54 (73.0) |
|  | Azithromycin | 20 (60.6) | 39 (52.7) |

Reviewer 3 had the following point:

1. The manuscript is quite good written (I noticed a little mistake at line 262; will instead of with).There are however some points which deserve more attention. First of all the tocilizumab group seems composed of a number of patients with more comorbidities (1,031 vs 0,7 pro patient) than controls and these subjects were not matched with controls on the basis of the specific comorbidity (diabetes with diabetes and so on). I naturally mean that the important thing at the end, among the two groups is that the single comorbidities are egually represented. There was also no mention of the other treatments undergone during hospital stay, and in my opinion it would be interesting to know which drugs have been administered in the two groups. It should be very good highlighted that in the tratment group, a low dose of Tocilizumab (400 mg once) was used.
   For what concerns the first point, I only would like to know authors opinion, in order to promote a positive exchange.
   Thankyou

Response: Great points.

1. The typo of “will” was changed to “with” – thank you!
2. The statement that we didn’t match specific comorbidities (e.g. diabetes) with specific comorbidities is correct. However, we did two sets of matching to balance the level of illness between groups. First, we matched on a set of variables, including number of comorbidities:
   - age
   - sex
   - body mass index (BMI)
   - ethnicity
   - number of comorbidities (hypertension, diabetes, coronary artery disease, liver disease, and chronic kidney disease)
   - presence of pulmonary disease
   - initial laboratory levels of C-reactive protein (CRP)
   - lactate dehydrogenase
   - initial calculated PaO2:FiO2
   - initial heart rate
   - level of care at admission

And second, we performed multivariable ordinal logistic regression models to determine the relationship between tocilizumab use and day 14 and day 28 ordinal score, adjusted for the ordinal score prior to medication administration, the ordinal score on day of hospital admission, and whether the subject was in the intensive care unit (ICU) at the time of medication administration.

Therefore, even though patients weren’t matched specifically for each comorbidity, they were matched for number of comorbidities, and numerous other disease severity markers (such as CRP and level of care at admission) and did a multivariable regression based on ordinal score.

3. The third point regarding concurrent medications is addressed above.
4. The last point regarding dose is a very good point and deserves more highlighting in the paper. We clarified the dose in several sections of the paper.
